# Growth Failure in Children with Congenital Heart Disease

**DOI:** 10.3390/children12050616

**Published:** 2025-05-09

**Authors:** Jihye Lee, Teresa Marshall, Harleah Buck, Mulder Pamela, Sandra Daack-Hirsch

**Affiliations:** 1 College of Nursing, The University of Iowa, Iowa City, IA 52242, USA; 2 The Saban Research Institute, Children’s Hospital Los Angeles, Los Angeles, CA 90027, USA; 3 Preventive and Community Dentistry, The University of Iowa, Iowa City, IA 52242, USA; teresa-marshall@uiowa.edu

**Keywords:** congenital heart disease, growth, failure to thrive, malnutrition, developmental, review

## Abstract

**Background/Objectives**: Growth failure is a common complication in children with congenital heart disease (CHD), yet its underlying mechanisms and consequences remain incompletely understood. This review aims to provide a comprehensive overview of growth failure in children with CHD and outline a framework of factors contributing to this condition. **Methods:** To lay the foundation for this narrative review, several databases were searched using broad search terms related to CHD and growth failure. **Results**: Growth failure is most pronounced during the first year of life, but often improves after achieving hemodynamic stability through surgical or medical interventions. However, children with complex conditions, such as single-ventricle physiology or multiple heart defects, may experience persistent growth impairment due to chronic disease effects. Specific features of CHD—cyanosis, pulmonary hypertension, and low cardiac output—can further hinder growth by disrupting endocrine function and impairing musculoskeletal development. Long-term use of medications and exposure to repeated diagnostic procedures also contribute to growth failure. Beyond physical effects, growth failure profoundly influences neurodevelopment, psychosocial well-being, and survival outcomes. Based on our review, we have developed a knowledge map to better understand the complexities of growth failure in children with CHD. **Conclusions**: A thorough understanding of the multifaceted contributors to growth failure in CHD is essential for identifying high-risk children and devising strategies to support optimal growth. Integrating this knowledge into clinical practice can improve long-term outcomes for children with CHD.

## 1. Introduction

Congenital heart disease (CHD) is a structural defect of the heart that is present at birth [1]. The structural and subsequent functional impairments in the heart often result in hemodynamic instability, creating unfavorable conditions for adequate physical growth and development [2,3,4]. Furthermore, CHD can lead to an imbalance between energy intake and expenditure, increasing the risk of growth failure [5,6]. Without timely and appropriate nutritional intervention, more than half of these children can develop severe growth failure [3,7]. Growth failure, defined as inadequate growth, an inability to maintain growth, or significantly decreased growth patterns [8,9], is closely associated with increased mortality and morbidity during infancy. It also adversely affects long-term functional development, including language, motor skills, intelligence, and psychosocial well-being [10,11,12,13,14].

Despite its clinical significance, the mechanisms linking CHD to growth failure are complex and not well understood. Because growth involves multiple interdependent physiological systems, individual studies often fail to capture the full complexity of the relationship between CHD and growth failure, potentially overlooking critical pathways and mechanisms. Understanding these mechanisms is essential for identifying patients at high risk for growth failure, detecting abnormal growth early, and delivering timely interventions to optimize growth trajectories.

This review describes the major disease characteristics of CHD and their role in the mechanisms that contribute to growth failure. It then details both the short- and long-term consequences of growth failure, highlighting the clinical implications for children with CHD. The purpose of this review is to provide a comprehensive overview of growth failure in children with CHD and to construct a knowledge map that explains the factors influencing growth failure in this population.

## 2. Methods

This review followed the methods proposed by Whittemore (2014) [15] and synthesizes findings from the previously published literature on growth failure in children with CHD. A narrative review is appropriate when existing quantitative studies differ methodologically and theoretically [16]. By evaluating and synthesizing multiple individual studies, this approach provides a comprehensive perspective that helps clarify what has been studied and broadens understanding of the current knowledge base.

A comprehensive literature search was conducted between April and May 2023 using the PubMed, CINAHL, MEDLINE, and Embase databases, without restrictions on the publication year. The search strategy included a combination of MeSH terms and free-text keywords to capture a broad range of relevant studies. The initial search focused on growth in children with CHD and included the following keywords: “growth” OR “growth disorder” OR “failure to thrive” OR “malnutrition” AND “heart defects, congenital” OR “congenital heart disease”. Additional keywords and free-text terms such as “growth” OR “failure to thrive” OR “malnutrition” AND “heart defect*” were searched in titles or abstracts to capture unindexed documents. In addition, to explore the genetic influences on growth failure in children with CHD, the search included terms such as “gene*” OR “chromosom*” combined with “congenital heart disease” OR “heart defects” AND “growth” OR “failure to thrive”. To explore the psychological consequences of growth failure, the search was expanded with terms like “growth” OR “failure to thrive” OR “malnutrition” AND “chronic disease” AND “child” OR “pediatric” AND “psycholog*” OR “depress*” OR “anxiety” OR “stress” OR “confiden*” OR “social.” More specific content for each topic was searched primarily using free-text keywords. Additionally, reference lists of identified articles were manually reviewed to ensure the comprehensiveness of the literature search.

The following inclusion criteria were applied: (a) studies published in English and available in full text; (b) original, peer-reviewed research articles; and (c) studies focused on children with congenital heart disease (CHD). In the subsequent data charting stage, relevant information from the selected studies was extracted and organized into a data chart. The final stage involved synthesizing and reporting the findings to construct a knowledge map of growth failure in children with CHD.

## 3. Results

The following section will review the literature on somatic growth, followed by discussions on genetic abnormalities, the pathophysiology of congenital heart disease (CHD), nutrition, and the implications of growth failure.

### 3.1. Somatic Growth in Children with Congenital Heart Disease

#### 3.1.1. Prenatal Growth

A fetus diagnosed with CHD is more than twice as likely to be small for gestational age or have a low birth weight [17,18,19]. Growth impairment is often associated with placenta dysfunction. While the relationships between these factors are complex and may be influenced by underlying genetic factors, placental dysfunction can restrict the connection between the fetus and the maternal environment [20]. The heart and placenta develop interdependently, sharing common signaling pathways and developing simultaneously. Placental dysfunction could contribute to cardiac abnormalities by hindering cardiac cell proliferation and angiogenic regulation [21], but it is also possible that placental dysfunction and CHD may arise concurrently or as a consequence of one another. Additionally, decreased mid-gestational placental blood flow due to placenta dysfunction can exacerbate fetal growth impairments [22]. Certain types of CHD, including hypoplastic left heart syndrome (HLHS), Tetralogy of Fallot (TOF), ventricular septal defect (VSD), and double outlet right ventricle (DORV), have been associated with low placental weight, which could contribute to small for gestational age at birth [23,24]. Moreover, fetuses with CHD are at a higher risk of premature birth (adjusted hazard ratio 2.1), which can lead to a reduction in the total number of cells at birth. Somatic growth occurs through an increase in cell number and cell size. Consequently, newborns with CHD are at increased risk for both low birth weight and being small for gestational age, which can heighten the likelihood of postnatal growth failure [24].

#### 3.1.2. Postnatal Growth

Growth failure in children with CHD is commonly observed after birth and can be categorized into three subtypes of growth failure: underweight, stunting, and wasting. Underweight refers to a low weight, stunting indicates a diminished height, and wasting signifies a low weight [25]. The World Health Organization (WHO) and Centers for Disease Control and Prevention (CDC) growth chart is the most widely used reference for assessing growth. A standardized score (z-score) of less than −2 standard deviations (SDs) or below the 5th percentile indicates growth failure (Table 1) [26].

In children with CHD, the prevalence and the dominant type of growth failure vary with age. This distinction is related to the difference between weight, which reflects short-term nutritional status, and height, indicative of chronic nutritional status [27]. During infancy, underweight status is more common [28], whereas stunting is less frequent but may suggest a prolonged period of impaired growth due to intrinsic and/or extrinsic factors [29].

Cardiac surgery is beneficial for growth outcomes. Although recovery rates vary, improvements in weight and height growth with age can be attributed to the impact of cardiac surgery. In a longitudinal study of children with Tetralogy of Fallot, anthropometric measurements were most impaired at the time of surgery (median age 1.6 years, mean weight for age z-score, mean WAZ: −1.04, *p* < 0.001, and mean height for age z-score, mean HAZ: −0.93, *p* < 0.001), and then gradually improved to no difference from the population average at follow-up (median age 14.2 years, mean WAZ: 0.16, *p* = 0.32, and mean HAZ: −0.05, *p* = 0.41) [30]. This finding suggests that surgical correction and medical intervention create an opportunity for catch-up growth, wherein the growth velocity accelerates after a preoperative growth delay [6,7,31,32].

In addition, the risk of growth failure varies with the severity and complexity of the disease. Simple defects or CHD with mild symptoms may not require medical attention. On the other hand, most cases of moderate to complex heart defects are accompanied by hemodynamic instability and require open-heart surgery. A study on growth failure in CHD demonstrated higher odds of being underweight and stunting in children requiring cardiac surgery (odds ratio, OR 12.1 and 3.7 for underweight and stunting, respectively) compared to those who did not (OR for underweight 3.9, OR for stunting 2.1) [33]. Another study also found that while being underweight and stunted were common across all diagnostic categories of CHD, the symptomatic CHD group—including conditions such as congestive heart failure and cyanosis, or both—had a significantly higher prevalence of underweight (79.6%) and stunting (81.3%) compared to the non-symptomatic group (14.8% for underweight and 11.1% for stunting) [34]. In some complex and single ventricle (SV) cases, cardiac surgery may not result in complete recovery of heart function, and subsequently, growth failure may persist into older childhood. Cohen [35] reported that children with SV still exhibited a low weight (mean WAZ: −0.49, 13.8% underweight) and height (mean HAZ: −1.15, 20.0% stunting, *p* < 0.01) even more than 4 years after undergoing the Fontan procedure. After the Fontan procedure, blood is passively directed to the lungs, and central venous pressure (CVP) serves as the driving force for pulmonary blood return. Both elevated and low CVP can result in various complications, such as protein-losing enteropathy (PLE), systemic venous collaterals, liver congestion, and impaired cardiac output [6,36,37]. These complications can increase energy expenditure and disrupt nutrient intake and absorption, raising the risk of growth failure. Consequently, children with SV defects are more prone to chronic growth failure [6,10,11,33,38]. Sandberg [39] reported that many patients with complex defects and SV, who experience stunting, do not attain normal height until adulthood. This highlights the need for ongoing monitoring and intervention to address growth failure in children with complex and SV defects.

#### 3.1.3. Genetic Abnormalities Linked to Congenital Heart Disease and Growth Failure

Although most patients with CHD do not have other anomalies, approximately 25 to 40% of CHD cases are related to genetic anomalies [40]. These genetic factors include abnormal chromosome numbers (aneuploidy), copy number variations (CNVs), and single gene defects. Aneuploidy accounts for a significant proportion of CHD, around 9 to 18% [41], and commonly observed anomalies, such as trisomy 21 and Turner syndrome, are often associated with short stature [42] (Table 2). CNVs, which refer to deletions or duplications of chromosomal segments, are implicated in 10–15% of CHD cases [43]. Conditions such as 22q11.2 deletion syndrome, Williams–Beuren syndrome, and Jacobsen syndrome, which result from CNVs, are frequently linked to a high incidence of CHD (50 to 90%) and growth failure [42,43]. Over the past decade, advances in molecular medicine have identified that certain syndromes are associated with a high prevalence of CHD. For example, the prevalence of CHD ranges from 50% to 85% in CHARGE syndrome, Costello syndrome, Noonan syndrome, and Kabuki syndrome, with these syndromes being significant contributors to phenotypic growth failure [42,44].

In addition to genetic anomalies linked to phenotypic growth failure, feeding difficulties in various syndromes—including CHARGE syndrome, Noonan syndrome, Williams–Beuren syndrome, and 22q11.2 deletion syndrome—endocrine complications, such as hypothyroidism and delayed puberty in Noonan syndrome and Williams–Beuren syndrome, and gastrointestinal issues in Jacobsen syndrome can further exacerbate growth failure [44,45]. While genetic factors are central to understanding growth failure in both fetal and postnatal contexts, it is essential to consider complex interactions.

**Table 2:** Chromosomal abnormalities associated with congenital heart disease (selected).

Syndrome	Gene	% CHD	Cardiac Phenotype	Other Clinical Findings	Reference
Chromosomal aneuploidy associated with CHD					
Trisomy 21 (Down syndrome)	Unknown	40–50%	AVSD, ASD, VSD, TOF, PDA	Short stature, cognitive deficits, immune system dysfunction, hypotonia, hypothyroidism	[46,47]
Turner syndrome (Monosomy X)	Unknown	25–35%	CoA, HLHS, BAV, AS	Short stature, cognitive deficits, lymphedema, webbed neck, primary amenorrhea	[48,49,50]
Copy number variations (CNVs)					
22q11.2 deletion (DiGeorge syndrome)	TBX1	75–85%	TOF, IAA, Truncus arteriosus, aortic arch anomalies, VSD, ASD	Growth retardation, growth hormone deficiency, skeletal abnormalities, hypothyroidism, hypoparathyroidism, immunodeficiency, hypocalcemia, distinctive facial features, feeding difficulties	[51,52]
Williams–Beuren syndrome	7q11.23 Deletion (ELN)	50–85%	SVAS, PPS, VSD, ASD	Dysmorphic facies, thick lips, growth abnormalities, short stature, connective tissue and skeletal abnormalities, intellectual disabilities, infantile hypercalcemia, renal disorders, hypothyroidism, feeding difficulties	[53,54]
Jacobsen syndrome	ETS1, FLI1	56%	HLHS, TOF, AS, VSD, CoA, Shone’s complex	Distinctive facial features, growth retardation, developmental delay, thrombocytopenia, strabismus, hammertoes	[55,56,57]
Single-gene variation					
CHARGE syndrome	CHD7	75–85%	TOF, PDA, DORV, AVSD, VSD	Coloboma, choanal atresia, genital and/or urinary hypoplasia, ear anomalies, growth retardation, developmental delay, intellectual disability	[58]
Costello syndrome	HRAS	44–52%	PS, ASD, VSD, HCM, arrhythmias	Short stature, feeding difficulties, developmental delay, broad facies, bitemporal narrowing, intellectual disability	[59]
Noonan syndrome	PTPN11, SOS1, RAF1, KRAS, NRAS, RIT1, SHOC2, SOS2, BRAF	75%	PS with dysplastic pulmonary valve, AVSD, TOF, ASD, VSD, PDA, HCM	Short stature, webbed neck, hypertelorism, pectus deformity, cryptorchidism, abnormal facies, ptosis, developmental delay	[60,61]
Kabuki syndrome	KMT2D, KDM6A	50%	CoA, VSD, TOF, TGA, HLHS	Growth deficiency, wide palpebral fissures, arched eyebrows, a flat or depressed nasal tip, large protuberant ears, intellectual disability, clinodactyly	[62,63]

Note. AS = aortic stenosis; ASD = atrial septal defects; AVSD = atrioventricular septal defect; CoA = coarctation of the aorta; DORV = double outlet of left ventricle; HCM = hypertrophic cardiomyopathy; HLHS = hypoplastic left heart syndrome; IAA = interruption of aorta; PDA = patent ductus arteriosus; PPS = peripheral pulmonary stenosis; PS = pulmonary stenosis; SVAS = supravalvular aortic stenosis; TGA = transposition of great arteries; TOF = Tetralogy of Fallot; VSD = ventricular septal defect.

### 3.2. Growth Challenges in Congenital Heart Disease

Physiological and structural abnormalities of the heart cause clinical symptoms, such as cyanosis, pulmonary artery hypertension (PAH), and heart failure. In addition to these symptoms, surgical correction and medications for CHD also affect energy absorption and the endocrine system, consequently impacting somatic growth. Thus, this section describes the major symptoms of CHD and the mechanisms that can affect growth.

#### 3.2.1. Low Oxygen Levels in Blood

One of the major critical manifestations of CHD is cyanosis, which occurs when the oxygen saturation in the blood is low [64]. Cyanosis is linked to heart defects, such as a right-to-left shunt that allows deoxygenation blood to enter the systemic circulation or a right heart obstructive disease that impedes sufficient blood oxygenation in the lungs, leading to hypoxemia [12]. Hypoxemia, in turn, leads to cellular hypoxia, triggering widespread physiological adaptations that negatively affect somatic growth [65]. In response to chronic hypoxia, the body activates hypoxia-inducible factors (HIFs), which mediate short-term cellular adaptation to low oxygen conditions. However, prolonged HIF activation can damage intestinal epithelial cells and disrupt mucosal integrity [66,67]. Additionally, hypoxia induces endoplasmic reticulum (ER) stress, which is associated with intestinal fibrosis and mucosal remodeling [68]. These hypoxia-driven alterations in the gastrointestinal tract compromise nutrient absorption, contributing to growth failure in children with CHD.

In addition, chronic hypoxemia in children with CHD stimulates erythropoiesis as a compensatory response to low oxygen levels. Although this increases red blood cell production, it also elevates iron demand, eventually depleting iron stores and resulting in iron-deficient erythropoiesis and anemia [69]. Studies report that up to 20–47% of children with CHD have iron deficiency anemia, with higher rates observed in those with cyanotic lesions [70,71]. This burden can be further exacerbated by the use of proton pump inhibitors (PPIs) and diuretics, which impair gastrointestinal iron absorption [72,73]. Anemia reduces oxygen delivery to peripheral tissues, increases metabolic stress, and impairs cellular growth, thereby contributing to both weight faltering and stunted linear growth [74,75,76].

Low oxygen levels can also affect the endocrine system, particularly the growth hormone (GH)–insulin-like growth factor I (IGF-I) axis. Chronic hypoxia diminished hepatic responsiveness to GH [5,65], reducing IGF-I secretion, which plays a critical role in stimulating musculoskeletal development and promoting longitudinal growth [77]. As a result, impaired GH-GIF I signaling can limit both muscle mass accrual and height gain, compounding the risk of growth failure in children with CHD.

#### 3.2.2. Kidney Dysfunction

In children with congenital heart disease (CHD), kidney dysfunction is recognized as a significant contributor to growth failure. Several mechanisms underline the increased risk of renal impairment in this population. Chronic hypoxemia, a hallmark of many CHD cases, leads to overstimulated erythropoiesis, which increases blood viscosity and may disrupt renal hemodynamics, including glomerular hyperfiltration [78]. Cyanotic nephropathy is reported in approximately 30–50% of children with cyanotic heart defects and is associated with impaired renal tubular function [79]. Beyond the chronic effects of hypoxemia, cardiac surgery involving cardiopulmonary bypass (CPB) is a well-recognized contributor to acute kidney injury (AKI). AKI occurs in approximately 30–40% of children undergoing CPB for congenital heart lesions, with rates reaching up to 60% in neonates [80,81,82].

In addition, several medications commonly used in the management of CHD have known nephrotoxic effects. For example, angiotensin-converting enzyme (ACE) inhibitors have been associated with renal impairment. A retrospective study of neonates with CHD demonstrated a significant decrease in renal function during ACE inhibitor therapy, with 42% developing AKI [83]. However, another retrospective review of children with CHD found no increased risk of AKI with ACE inhibitors, even when used in combination with furosemide [84]. Given the lack of consensus regarding the effects of ACE inhibitors on renal function, it is challenging to generalize these findings to all patients with congenital heart disease.

Kidney dysfunction, whether stemming from pathophysiological alterations, surgery, or medication, can exacerbate growth failure in children with CHD. Uremia, chronic inflammation, and metabolic acidosis, frequently observed in chronic kidney disease, impair protein synthesis and suppress appetite, contributing to protein–energy wasting [85,86,87]. Additionally, kidney dysfunction contributes to growth hormone resistance by reducing the density of GH receptors, impairing GH-activated post-receptors, and decreasing levels of free IGF-I due to increased inhibitory IGF-binding proteins [88]. These disruptions result in both poor weight gain and linear growth. Thus, in children with CHD, kidney dysfunction can be a critical modifier of growth failure.

#### 3.2.3. Pulmonary Hypertension

Pulmonary hypertension (PH) can develop as a secondary complication of CHD due to increased pulmonary blood flow or elevated pulmonary vascular resistance (PVR) [89]. PH is commonly associated with CHD involving left-to-right shunts, such as atrial septal defect (ASD), ventricular septal defect (VSD), and patent ductus arteriosus (PDA), as well as left heart obstructive conditions, including aortic and mitral valve abnormalities. This section will focus specifically on secondary pulmonary hypertension resulting from CHD.

Numerous studies have demonstrated the negative impact of PH on somatic growth [90,91,92]. In a study on symptomatic CHD, children with PH were twice as likely to experience growth failure compared to those without PH (OR 2.01, 95% CI: 1.13–3.59, *p* < 0.05) [93]. Similarly, Woldesenbet et al. [94] reported that PH increased the risk of being underweight by 1.885 times (OR 1.885, 95% CI: 1.094–3.246, *p* = 0.022). In PH, elevated pulmonary pressure induces stress on the pulmonary capillary walls, increasing capillary permeability. This leads to the leakage of plasma and other components into the surrounding lung tissue, causing pulmonary congestion [95]. If untreated, PH can lead to vascular remodeling, including medial wall hypertrophy, intimal fibrosis, and changes in the pulmonary vascular bed, ultimately resulting in increased PVR. As PH progresses, gas exchange in the alveoli becomes impaired, and in some cases, this can lead to an irreversible condition known as Eisenmenger syndrome, which is accompanied by severe right heart failure [95,96].

The symptoms and severity of PH largely depend on the size of the shunt, and approximately 80% of patients experience dyspnea, fatigue, and significant exercise limitations [97]. These symptoms are often associated with poor appetite, feeding intolerance, and an imbalance between energy intake and expenditure, further exacerbating the risk of growth failure [92,98]. The systemic effects of PH affect the body’s ability to utilize nutrients effectively, complicating the management of growth failure in these patients. Effective management requires addressing both the cardiac and pulmonary dysfunctions associated with PH, as well as supporting nutritional needs through more than just increasing feed intake [31].

#### 3.2.4. Effects on Growth-Related Hormone

CHD can impact on the endocrine systems involved in somatic growth in several ways. One hypothesis is that impaired heart function reduces oxygen supply to vital organs, including the liver, which plays a crucial role in the secretion and regulation of IGF-I [4,5,65,97]. IGF-I is essential for both (a) longitudinal bone growth by promoting the differentiation and hypertrophy of chondrocytes in the growth plate and (b) body weight growth by influencing the division of adipocyte precursor cells [99]. Low levels of IGF-I have been reported in children with cyanotic heart defects, suggesting that hypoxia may negatively affect the synthesis and secretion of growth-related hormone, including IGF-I [4,5,100]. Additionally, the Fontan procedure, commonly performed for SV physiology, may increase the risk of growth failure. In the Fontan circulation, blood moves passively without the assistance of the right ventricle, which can lead to venous stagnation [101] and, over time, liver congestion and cirrhosis [37]. The liver not only synthesizes IGF-I but is also responsible for converting thyroxine (T4) into triiodothyronine (T3), a thyroid hormone that regulates metabolic rate, digestive functions, and the maintenance of muscle and bone tissue [102,103]. Therefore, Fontan-associated liver disease (FALD) can limit the synthesis and secretion of both IGF-I and T3, compounding the risk of growth failure.

Moreover, nutritional status plays a crucial role in regulating growth hormones, contributing to the risk of growth failure. Weight loss before and after surgery is common in children with CHD [2,3,75,104], and surgical complications such as protein-losing enteropathy further increase the risk of protein malnutrition. Insulin-like growth factor I (IGF-I) synthesis is highly sensitive to nutritional status, and since both growth hormone (GH) and IGF-I circulate in protein-bound form, malnutrition—particularly protein deficiency—can impair their activation, further compromising growth [102,103,105].

Beyond IGF-I and GH, other endocrine and systemic factors can contribute to growth failure in CHD. Cortisol, a glucocorticoid released in response to physical and psychological stress, is critical in regulating metabolism, immune function, and inflammatory responses. In children with CHD, both chronic and acute stressors, including chronic hypoxia, repeated hospitalizations, invasive procedures, and major surgeries, can lead to sustained elevations in cortisol levels [106]. It promotes muscle protein catabolism, suppresses GH secretion and IGF-I production, and impairs tissue responsiveness to growth signals [107,108]. Additionally, elevated cortisol disrupts bone metabolism by inhibiting osteoblast activity and promoting osteoclast-mediated resorption, which may further contribute to linear growth failure [106,109]. In the perioperative period, the heightened stress response following cardiopulmonary bypass surgery can exacerbate these effects, especially in neonates and infants with limited physiological reserves.

Chronic inflammation, often seen in children with CHD, also contributes to growth failure. Elevated levels of inflammatory cytokines, such as tumor necrosis factor-alpha (TNF-α) and interleukin-6 (IL-6) [110,111,112], directly impact growth regulation [113]. These cytokines can suppress GH secretion from the pituitary gland, leading to a reduction in IGF-I production by the liver. Additionally, TNF-α and IL-6 interfere with IGF-I signaling by disrupting the IGF-I receptor pathway, limiting its effectiveness in promoting cell growth and differentiation in bones and tissues [114]. Together, these factors—impaired hormonal signaling, poor nutrition, and muscle loss—can create a cycle that exacerbates growth failure in children with CHD.

Thyroid function is another key aspect of growth regulation. Children with CHD often experience thyroid dysfunction due to the embryonic and genetic coexistence between the heart and the thyroid gland [115,116]. Additionally, cardiac surgeries with cardiopulmonary bypass can lead to nonthyroidal illness syndrome (NTIS) [117]. NTIS is characterized by abnormal thyroid hormone levels, which often remain suppressed even beyond the critical postoperative period [118]. Hypothyroidism, whether primary or secondary to CHD, can further contribute to growth failure. Low levels of thyroid hormones reduce metabolic rate, impair normal growth and developmental processes, and inhibit bone growth, leading to delayed skeletal maturation and growth retardation [119,120].

The contribution of various endocrine and systemic factors to growth failure in children with congenital heart disease is multifaceted. In addition to IGF-I and growth hormone, other factors, such as renal function and iron deficiency, which were not covered in this review, should also be considered to fully understand the complexity of growth failure in CHD.

### 3.3. Nutrition in Children with Congenital Heart Disease

#### 3.3.1. Malnutrition in Children with Congenital Heart Disease

Malnutrition is a common concern among children with congenital heart disease (CHD), with an estimated 15–60% affected by moderate to severe malnutrition [2,3,75,121]. This wide variation in prevalence may be related to factors such as regional differences, age, and specific CHD diagnoses. Beyond these external influences, malnutrition is often driven by insufficient energy intake, increased metabolic demands, frequent hospitalizations, and prolonged illness due to the disease [75,122]. Infants with CHD experience high energy expenditure during feeding, leading to fatigue and reduced appetite, which further limits their overall energy intake [31]. Studies indicate that 20–30% of children with CHD consume fewer calories than recommended, with protein intake averaging only 70–80% of the recommended daily amount [123,124,125].

In terms of metabolic rate, children with CHD have a higher resting energy expenditure (REE) than their peers without CHD [12,123,126,127]. Additionally, low-birth-weight infants with CHD demonstrate elevated oxygen consumption (10.9 ± 1.4 mL/min/kg) compared to normal-weight infants with CHD (7.5 ± 2.0 mL/min/kg) [128]. These findings indicate that children with CHD may experience increased metabolic demands, potentially contributing to malnutrition.

Additionally, prolonged and recurrent hospitalizations, along with complications related to surgical intervention, contribute to both acute and chronic malnutrition in children with CHD [90,104,129,130]. For example, therapeutic feeding restrictions and extended fasting periods increase the risk of acute malnutrition [131]. A study assessing the nutritional status of children immediately after cardiac surgery found that 50% of children with CHD experienced acute protein malnutrition, and 40% suffered from chronic protein malnutrition [124]. Furthermore, surgical complications such as chylothorax, vocal cord paralysis, diaphragm palsy, and protein-losing enteropathy (PLE) further heighten the risk of both acute and chronic malnutrition [132]. Consequently, nearly one-third of children with CHD may experience malnutrition following surgical procedures [91,122,133]. In summary, children with CHD are highly susceptible to malnutrition, highlighting the need for closer monitoring and evaluation of their nutritional status.

#### 3.3.2. Nutritional Intervention for Children with Congenital Heart Disease

Heart failure, prolonged ventilator or sedative use, and post-operative complications, such as gastroesophageal reflux and vocal cord injury, can contribute to feeding difficulties and insufficient caloric intake in children with CHD [91,122,132,133]. Although studies suggest that preoperative feeding can improve postoperative feeding tolerance [134,135], there remains clinical debate regarding the timing and safety of initiating enteral feeding in hemodynamically unstable infants. Nonetheless, if an infant with CHD is hemodynamically stable, gradual advancement of enteral feeds may be initiated under close monitoring for signs of feeding intolerance [136]. For high-risk infants, enteral nutrition continues to be considered beneficial, although larger enteral volumes (e.g., >100 mL/kg/day) have been associated with increased necrotizing enterocolitis (NEC) risk [137]. There is still no consensus on the optimal volume and rate of advancement, making individualized care essential.

Determining accurate energy requirements is challenging due to individual variations in metabolic responses and energy needs. Despite these challenges, providing optimal calories and protein in the postoperative phase is crucial in managing metabolic changes induced by surgical stress. Immediately after surgery, catabolic hormones are released, increasing protein and fat consumption to support wound healing [138]. This catabolic process occurs primarily in peripheral tissues to support wound healing. In the subsequent anabolic phase, muscle mass and body fat are gradually restored through protein synthesis, and the transition from catabolism to anabolism depends on adequate nutrient supply [139]. To meet these demands, current recommendations suggest high-caloric feeding strategies in the postoperative period, gradually increasing to 120–150 kcal/kg/day, and up to 150–170 kcal/kg/day in cases of severe weight loss [140,141]. However, while increased caloric intake is necessary to support recovery, it must be balanced against the risk of feeding intolerance, particularly in vulnerable infants. Randomized controlled trials (RCTs) comparing postoperative outcomes between high-energy and standard formulas have shown that infants receiving high-energy formulas experienced a higher incidence of feeding intolerance, including diarrhea, gastric retention, increased gastric residual volume, gastric bleeding, and emesis [142,143]. Simultaneously, protein intake should be increased to meet heightened needs. A minimum protein intake of 1.1 g/kg/day, or approximately 1.5 to 2 times the standard requirement, is recommended and may be increased up to 3 g/kg/day when clinically indicated [136,141,144]. A protein–energy ratio of 9–12% is considered optimal to reduce protein loss. However, in practice, achieving these targets remains a challenge. Toole [124] reported that only 68% of children with CHD met their protein and energy requirements postoperatively. Current nutrition guidelines suggest 40–50% of total calories from carbohydrates, 7–16% from protein, and 34–35% from fats to meet the energy and metabolic needs of infants with CHD [136].

Energy expenditure in children with CHD varies due to multiple factors, such as preoperative nutritional status, the use of sedatives, vasoconstrictive medications, and mechanical ventilation. Despite these variations, postoperative REE is typically elevated (estimated at 39–73 kcal/kg/day), often exceeding that of healthy children [27,145,146]. Given the increased metabolic demand, individualized caloric prescriptions are necessary, but no universal formula exists, and practice patterns vary considerably across institutions.

When sufficient oral intake is not achieved, healthcare providers may consider enteral nutrition to support nutritional status and somatic growth. However, a multicenter study reported that only 53% of children with CHD, who showed delayed growth, received enteral nutrition in the preoperative phase, indicating that many children are not receiving adequate nutritional support preoperatively [134]. Both pre-and postoperative enteral nutrition have been associated with reduced postoperative mortality and improvements in nutritional outcomes, including weight gain and serum albumin levels [147,148,149]. Still, barriers remain regarding timely implementation, including inconsistent guidelines, lack of specialized nutrition teams, and concerns about fluid restrictions in the perioperative period [150]

Additionally, careful attention to micronutrient supplementation is essential in children with CHD due to increased risk of deficiencies from diuretic use, prolonged parenteral nutrition, and surgical stress. Key micronutrients such as zinc, magnesium, and calcium play critical roles in immune function, cardiovascular regulation, and bone health [151,152,153]. Regular monitoring of laboratory values and timely supplementation can help support recovery and promote nutritional status. According to the guidance of the European Society for Pediatric Gastroenterology, Hepatology, and Nutrition (ESPGHAN), electrolyte administration can be initiated from the first day of life in infants weighing less than 5 kg, guided by their serum electrolyte levels. Table 3 summarizes electrolyte requirements for critically ill neonates [154].

In summary, nutritional intervention can help improve clinical outcomes and optimize somatic growth in children with CHD. Active, individualized nutritional interventions are needed to meet each child’s energy and nutrient requirements, accompanied by regular, continuous monitoring of nutritional and growth status.

### 3.4. Clinical and Psychological Aspects of Growth in Congenital Heart Disease

Somatic growth or growth failure can be a significant predictor of short- and long-term prognosis of disease [155,156]. In terms of short-term outcomes, underweight and malnutrition are risk factors for mortality in children with CHD [157]. For instance, surgical mortality risk in normal-weight neonates with CHD is 5.7%, while it rises to 8.6% in low-weight neonates [155]. A study of infants with HLHS found that higher weight was associated with shorter durations of mechanical ventilation (r = −0.47, *p* < 0.001), ICU stays (r = −0.41, *p* = 0.006), and total hospital stays (r = −0.46, *p* = 0.002) [90]. Furthermore, malnutrition, especially protein malnutrition, increases the risk of postoperative infection and delays wound healing, which negatively impacts postoperative outcomes and raises medical costs [34,124,155,158].

In the long term, a child’s growth status is a good predictor of future development and functional well-being [7,159]. Growth failure early in life can adversely affect language and gross motor development [7]. One study examining mental and psychomotor development in children with CHD found that low height and head circumference z-scores correlated with lower mental development, while low weight and height z-scores were linked to reduced psychomotor development [160]. Another study reported that 23% of children with CHD experienced developmental delays in at least one domain—cognitive, language, and motor skills—and 21% of children showed delays in two or more areas. These delays were linked to poor linear growth, a history of tube feeding, and extended hospital stays [161]. Additionally, factors, such as prolonged ICU stays, use of sedatives, and recurrent hospitalization can further contribute to the developmental challenges [162].

Growth failure can also negatively impact psychosocial development, likely due to social perception of height and body image. Height is traditionally seen as a socially desirable attribute [163], with taller individuals often perceived as more capable and attractive across many cultures [159,164]. This perception extends to childhood, where children who are shorter and thinner than their peers often report lower body confidence [14]. For children with CHD, additional factors, including surgical scars and limited physical capabilities, may further harm body image [165,166]. Children who view their bodies negatively are likely to feel unpopular and unhappy, which can increase social isolation and reduce social engagement [167,168]. Consequently, growth failure is associated with poorer clinical outcomes and diminished psychosocial functioning. Therefore, addressing growth failure in children should include psychological support alongside proactive nutritional interventions.

Importantly, growth failure and its associated consequences often extend into adolescence and adulthood. Individuals with complex CHD or other coexisting conditions may continue to experience growth deficits, altered body composition, and delayed pubertal development [169]. Therefore, long-term strategies for monitoring and supporting these patients are essential. Effective transition planning and adult-focused follow-up care are critical components of this process [170]. Adult congenital heart disease (ACHD) clinics should routinely assess patients’ growth history, nutritional status, bone health, and psychosocial well-being, particularly during the transition from pediatric to adult care.

## 4. Discussion

**Figure 1:**
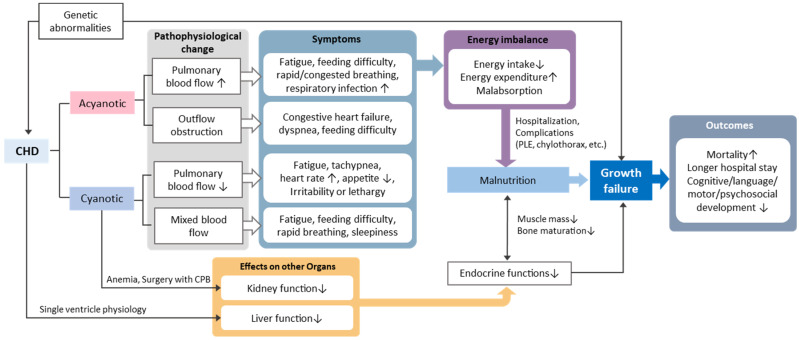
Knowledge map for growth failure in children with congenital heart disease. Note. CHD = congenital heart disease; CPB = cardiopulmonary bypass; PLE = protein loss enteropathy.

The purpose of this review was to provide a comprehensive overview of growth failure in children with CHD and to construct a knowledge map outlining the factors influencing growth failure in this population (Figure 1). While this objective was addressed in the previous section, several points warrant further discussion.

In terms of utility, we expect that the knowledge map serves as a valuable tool for various stakeholders. Clinicians may use it to guide personalized care plans and improve early identification of at-risk children, while researchers can use it as a framework for hypothesis generation and study design based on the complex interactions depicted.

As demonstrated throughout this review, growth failure is a multifaceted phenomenon. Therefore, addressing growth failure in chronically ill children, particularly those with CHD, requires a multidisciplinary care team. Coordinated efforts among pediatric cardiologists, dietitians, endocrinologists, genetic counselors, psychologists, and rehabilitation therapists are essential for providing comprehensive, individualized care. Each specialty can bring distinct expertise, ranging from optimizing perioperative nutrition and managing feeding difficulties to evaluating endocrine dysfunction, supporting neurodevelopment, and promoting psychological well-being. A team-based approach enables timely, integrated interventions that can significantly enhance growth outcomes and overall quality of life.

Nonetheless, potential limitations must be considered. First, the map may fail to account for emerging contributors to growth failure not yet widely captured in the literature and may oversimplify the diverse and dynamic nature of patient experience. As scientific knowledge advances, the map should be updated to incorporate new findings, ensuring its ongoing relevance and utility. Furthermore, the map may not fully reflect the unique challenges faced by patients with CHD, particularly in relation to psychosocial factors. Incorporating patient-reported outcomes in future could provide more robust evidence on psychosocial outcomes. Second, many of the studies cited in this review are observational or retrospective in design, limiting causal inference. Future studies should consider standardized methodologies based on RCTs to enhance the rigor and applicability of the results.

Looking ahead, several critical research gaps warrant targeted investigation. First, syndrome-specific growth management strategies are urgently needed for children with coexisting genetic and cardiac conditions, such as 22q11.2 deletion syndrome or Turner syndrome, who often experience compounded growth impairments due to both endocrine abnormalities and CHD. Tailored interventions, including growth hormone therapy, nutritional support, and psychosocial care, remain insufficiently explored in these high-risk groups. Second, there is a notable lack of robust, longitudinal research evaluating the long-term effectiveness of multidisciplinary interventions. These should not only target somatic growth but also consider broader outcomes such as neurodevelopment, academic achievement, and quality of life. Future studies should prioritize longitudinal designs with extended follow-up into adolescence and adulthood to understand the lasting impact of early and sustained interventions for growth failure on the well-being of children with CHD.

## 5. Conclusions

CHD is an umbrella term covering a range of diagnoses, each with different hemodynamic characteristics. To understand growth failure in CHD, it is essential to consider these specific disease characteristics rather than the CHD diagnosis alone. The hemodynamic changes associated with CHD disrupt energy balance and impact the endocrine and musculoskeletal systems, increasing the risk of growth failure. This growth failure has significant short-term implications, including increased mortality rates, prolonged hospital stays, and higher infection susceptibility. Long-term effects include impacts on language development, motor skills, and psychosocial well-being. Early recognition and intervention for abnormal growth patterns can significantly improve somatic growth in the future. To enhance both clinical outcomes and quality of life, further research is needed to identify effective monitoring strategies and interventions to prevent or mitigate the effects of growth failure in children with CHD.

## Figures and Tables

**Table 1:** Determinants of underweight, stunting, and wasting.

Z-Score (%) Cut-Off (WHO Growth Chart)	Percentile Cut-Off (CDC Growth Chart)	Weight for Age (WAZ)	Height for Age (HAZ)	Weight for Height (WHZ)	Body Mass Index (BMI) for Age
≥+3 (99%)	≥95th	-	-	Obese	Obese
≥+2 (97%)	≥85th and <95th			Overweight	Overweight
≥+1 (85%)		Normal	Normal	Possible risk of overweight	
<−1 (15%)		Slightly underweight	Slightly stunted	Slightly wasted	
<−2 (3%)	<5th	Underweight	Stunted	Wasted	Underweight
<−3 (1%)		Severely underweight	Severely stunted	Severely wasted	

Note. WHO = World Health Organization; CDC = Centers for Disease Control and Prevention; WAZ = weight for age z-score; HAZ = height for age z-score; WHZ = weight for height z-score.

**Table 3:** Electrolyte requirements for critically ill neonates (under 5 kg).

Electrolytes	Day 1	Day 2	Day 3	Day 4	Day 5	Intermediate Phase
Sodium (mmol/kg/d)	0–2	0–2	0–2	1–3	1–3	2–3
Potassium (mmol/kg/d)	0–3	0–3	0–3	2–3	2–3	1–3
Calcium (mmol/kg/d)	0.8–1.5	0.8–1.5	0.8–1.5	0.8–1.5	0.8–1.5	0.5
Chloride (mmol/kg/d)	0–3	0–3	0–3	2–5	2–5	2–3

## Data Availability

Data sharing is not applicable.

## Abbreviations

The following abbreviations are used in this manuscript:

CHD	Congenital heart disease
WAZ	Weight for age z-score
HAZ	Height for age z-score
WHZ	Weight for height z-score
PH	Pulmonary hypertension

## References

[1] Micheletti A. (2019). Congenital heart disease classification, epidemiology, diagnosis, treatment, and outcome. Congenital Heart Disease.

[2] Blasquez A., Clouzeau H., Fayon M., Mouton J., Thambo J., Enaud R., Lamireau T. (2016). Evaluation of nutritional status and support in children with congenital heart disease. Eur. J. Clin. Nutr..

[3] El-Koofy N., Mahmoud A.M., Fattouh A.M. (2017). Nutritional rehabilitation for children with congenital heart disease with left to right shunt. Turk. J. Pediatr..

[4] Harrison J.F., Shingleton A.W., Callier V. (2015). Stunted by developing in hypoxia: Linking comparative and model organism studies. Physiol. Biochem. Zool..

[5] Dinleyici E.C., Kilic Z., Buyukkaragoz B., Ucar B., Alatas O., Aydogdu S.D., Dogruel N. (2007). Serum IGF-1, IGFBP-3 and growth hormone levels in children with congenital heart disease: Relationship with nutritional status, cyanosis and left ventricular functions. Neuroendocrinol. Lett..

[6] Vogt K.N., Manlhiot C., Van Arsdell G., Russell J.L., Mital S., McCrindle B.W. (2007). Somatic growth in children with single ventricle physiology: Impact of physiologic state. J. Am. Coll. Cardiol..

[7] Chen C.W., Li C.Y., Wang J.K. (2007). Growth and development of children with congenital heart disease. J. Adv. Nurs..

[8] Cole S.Z., Lanham J.S. (2011). Failure to thrive: An update. Am. Fam. Physician.

[9] Monteiro F.P.M., Araujo T.L.D., Cavalcante T.F., Leandro T.A., Filho S.P.C.S. (2016). Child growth: Concept analysis. Texto Contexto-Enferm..

[10] Aguilar D.C., Raff G.W., Tancredi D.J., Griffin I.J. (2015). Childhood growth patterns following congenital heart disease. Cardiol. Young.

[11] Cohen M.S., Zak V., Atz A.M., Printz B.F., Pinto N., Lambert L., Pemberton V., Li J.S., Margossian R., Dunbar-Masterson C. (2010). Anthropometric measures after Fontan procedure: Implications for suboptimal functional outcome. Am. Heart J..

[12] Irving S.Y., Medoff-Cooper B., Stouffer N.O., Schall J.I., Ravishankar C., Compher C.W., Marino B.S., Stallings V.A. (2013). Resting energy expenditure at 3 months of age following neonatal surgery for congenital heart disease. Congenit. Heart Dis..

[13] Lambert L.M., McCrindle B.W., Pemberton V.L., Hollenbeck-Pringle D., Atz A.M., Ravishankar C., Campbell M.J., Dunbar-Masterson C., Uzark K., Rolland M. (2020). Longitudinal study of anthropometry in Fontan survivors: Pediatric heart network Fontan study. Am. Heart J..

[14] Nousi D., Christou A. (2010). Factors affecting the quality of life in children with congenital heart disease. Health Sci. J..

[15] Whittemore R., Chao A., Jang M., Minges K.E., Park C. (2014). Methods for knowledge synthesis: An overview. Heart Lung.

[16] Paré G., Kitsiou S. (2017). Methods for literature reviews. Handbook of eHealth Evaluation: An Evidence-Based Approach.

[17] Ghanchi A., Derridj N., Bonnet D., Bertille N., Salomon L.J., Khoshnood B. (2020). Children born with congenital heart defects and growth restriction at birth: A systematic review and meta-analysis. Int. J. Environ. Res. Public Health.

[18] Sochet A.A., Ayers M., Quezada E., Braley K., Leshko J., Amankwah E.K., Quintessenza J.A., Jeffrey P., Dadlani G. (2013). The importance of small for gestational age in the risk assessment of infants with critical congenital heart disease. Cardiol. Young.

[19] Wallenstein M., Harper L., Odibo A., Roehl K., Macones G., Cahill A. (2011). Fetal congenital heart disease and intrauterine growth restriction: A retrospective cohort study. Am. J. Obstet. Gynecol..

[20] Brown L.D., Hay W.W. (2016). Impact of placental insufficiency on fetal skeletal muscle growth. Mol. Cell. Endocrinol..

[21] Limperopoulos C., Wessel D.L., Plessis A.J.D. (2022). Understanding the Maternal-Fetal Environment and the Birth of Prenatal Pediatrics. J. Am. Heart Assoc..

[22] Ho D.Y., Josowitz R., Katcoff H., Griffis H.M., Tian Z., Gaynor J.W., Rychik J. (2020). Mid-gestational fetal placental blood flow is diminished in the fetus with congenital heart disease. Prenat. Diagn..

[23] Jones H.N., Olbrych S.K., Smith K.L., Cnota J.F., Habli M., Ramos-Gonzales O., Owens K.J., Hinton A.C., Muglia L.J., Hinton R.B. (2015). Hypoplastic left heart syndrome is associated with structural and vascular placental abnormalities and leptin dysregulation. Placenta.

[24] Matthiesen N.B., Østergaard J.R., Hjortdal V.E., Henriksen T.B. (2021). Congenital heart defects and the risk of spontaneous preterm birth. J. Pediatr..

[25] Miller B.S., Johnson D.E., Kang J.E., Petryk A. (2012). Growth Failure in International Adoptees. Handbook of Growth and Growth Monitoring in Health and Disease.

[26] Khadilkar V., Khadilkar A. (2011). Growth charts: A diagnostic tool. Indian J. Endocrinol. Metab..

[27] Mehta N.M., Corkins M.R., Lyman B., Malone A., Goday P.S., Carney L., Monczka J.L., Plogsted S.W., Schwenk W.F. (2013). Defining pediatric malnutrition: A paradigm shift toward etiology-related definitions. J. Parenter. Enter. Nutr..

[28] Zhang M., Wang L., Huang R., Sun C., Bao N., Xu Z. (2020). Risk factors of malnutrition in Chinese children with congenital heart defect. BMC Pediatr..

[29] Radman M., Mack R., Barnoya J., Castañeda A., Rosales M., Azakie A., Mehta N., Keller R., Datar S., Oishi P. (2014). The effect of preoperative nutritional status on postoperative outcomes in children undergoing surgery for congenital heart defects in San Francisco (UCSF) and Guatemala City (UNICAR). J. Thorac. Cardiovasc. Surg..

[30] Cheung M.M., Davis A.M., Wilkinson J.L., Weintraub R.G. (2003). Long term somatic growth after repair of tetralogy of Fallot: Evidence for restoration of genetic growth potential. Heart.

[31] Forchielli M.L., McColl R., Walker W.A., Lo C. (1994). Children with congenital heart disease: A nutrition challenge. Nutr. Rev..

[32] Hapuoja L., Kretschmar O., Rousson V., Dave H., Naef N., Latal B. (2021). Somatic growth in children with congenital heart disease at 10 years of age: Risk factors and longitudinal growth. Early Hum. Dev..

[33] Daymont C., Neal A., Prosnitz A., Cohen M.S. (2013). Growth in children with congenital heart disease. Pediatrics.

[34] Cameron J.W., Rosenthal A., Olson A.D. (1995). Malnutrition in hospitalized children with congenital heart disease. Arch. Pediatr. Adolesc. Med..

[35] Cohen M.I., Bush D.M., Ferry R.J., Spray T.L., Moshang T., Wernovsky G., Vetter V.L. (2000). Somatic growth failure after the Fontan operation. Cardiol. Young.

[36] Rychik J. (1995). Protein-losing enteropathy after Fontan operation. Congenit. Heart Dis..

[37] Gordon-Walker T.T., Bove K., Veldtman G. (2019). Fontan-associated liver disease: A review. J. Cardiol..

[38] Burch P.T., Gerstenberger E., Ravishankar C., Hehir D.A., Davies R.R., Colan S.D., Sleeper L.A., Newburger J.W., Clabby M.L., Williams I.A. (2014). Longitudinal assessment of growth in hypoplastic left heart syndrome: Results from the single ventricle reconstruction trial. J. Am. Heart Assoc..

[39] Sandberg C., Rinnström D., Dellborg M., Thilén U., Sörensson P., Nielsen N.-E., Christersson C., Wadell K., Johansson B. (2015). Height, weight and body mass index in adults with congenital heart disease. Int. J. Cardiol..

[40] Bernstein D. (2004). Evaluation of the cardiovascular system. Nelson Textb. Pediatr..

[41] Hartman R.J., Rasmussen S.A., Botto L.D., Riehle-Colarusso T., Martin C.L., Cragan J.D., Shin M., Correa A. (2011). The contribution of chromosomal abnormalities to congenital heart defects: A population-based study. Pediatr. Cardiol..

[42] Yasuhara J., Garg V. (2021). Genetics of congenital heart disease: A narrative review of recent advances and clinical implications. Transl. Pediatr..

[43] Ehrlich L., Prakash S.K. (2022). Copy-number variation in congenital heart disease. Curr. Opin. Genet. Dev..

[44] Pierpont M.E., Brueckner M., Chung W.K., Garg V., Lacro R.V., McGuire A.L., Mital S., Priest J.R., Pu W.T., Roberts A. (2018). Genetic basis for congenital heart disease: Revisited: A scientific statement from the American Heart Association. Circulation.

[45] Zaidi S., Brueckner M. (2017). Genetics and genomics of congenital heart disease. Circ. Res..

[46] Bittles A.H., Bower C., Hussain R., Glasson E.J. (2007). The four ages of Down syndrome. Eur. J. Public Health.

[47] Allen H.D., Driscoll D.J., Shaddy R.E., Feltes T.F. (2013). Moss & Adams’ Heart Disease in Infants, Children, and Adolescents: Including the Fetus and Young Adult.

[48] Sybert V.P. (1998). Cardiovascular malformations and complications in Turner syndrome. Pediatrics.

[49] Guarneri M.P., Abusrewil S.A., Bernasconi S., Bona G., Cavallo L., Cicognani A., Di B.E., Salvatoni A. (2001). Turner’s syndrome. J. Pediatr. Endocrinol. Metab..

[50] Gravholt C.H., Andersen N.H., Christin-Maitre S., Davis S.M., Duijnhouwer A., Gawlik A., Gawlik A., Maciel-Guerra A.T., Gutmark-Little I., Fleischer K. (2024). Clinical practice guidelines for the care of girls and women with Turner syndrome: Proceedings from the 2023 Aarhus International Turner Syndrome Meeting. Eur. J. Endocrinol..

[51] Christiansen J., Dyck J.D., Elyas B.G., Lilley M., Bamforth J.S., Hicks M., Sprysak K.A., Tomaszewski R., Haase S.M., Vicen-Wyhony L.M. (2004). Chromosome 1q21. 1 contiguous gene deletion is associated with congenital heart disease. Circ. Res..

[52] Bernier R., Steinman K.J., Reilly B., Wallace A.S., Sherr E.H., Pojman N., Mefford H.C., Gerdts J., Earl R., Hanson E. (2016). Clinical phenotype of the recurrent 1q21. 1 copy-number variant. Genet. Med..

[53] Morris C.A., Lenhoff H.M., Wang P.P. (2006). Williams-Beuren Syndrome: Research, Evaluation, and Treatment.

[54] Pober B.R. (2010). Williams–Beuren syndrome. N. Engl. J. Med..

[55] Jacobsen P., Hauge M., Henningsen K., Hobolth N., Mikkelsen M., Philip J. (1973). An (11; 21) translocation in four generations with chromosome 11 abnormalities in the offspring: A clinical, cytogenetical, and gene marker study. Hum. Hered..

[56] Favier R., Akshoomoff N., Mattson S., Grossfeld P. (2015). Jacobsen syndrome: Advances in our knowledge of phenotype and genotype. Am. J. Med. Genet. Part C Semin. Med. Genet..

[57] Grossfeld P.D., Mattina T., Lai Z., Favier R., Jones K.L., Cotter F., Jones C. (2004). The 11q terminal deletion disorder: A prospective study of 110 cases. Am. J. Med. Genet. Part A.

[58] Trider C.L., Arra-Robar A., van Ravenswaaij-Arts C., Blake K. (2017). Developing a CHARGE syndrome checklist: Health supervision across the lifespan (from head to toe). Am. J. Med. Genet. Part A.

[59] Lin A.E., Alexander M.E., Colan S.D., Kerr B., Rauen K.A., Noonan J., Baffa J., Hopkins E., Sol-Church K., Limongelli G. (2011). Clinical, pathological, and molecular analyses of cardiovascular abnormalities in Costello syndrome: A Ras/MAPK pathway syndrome. Am. J. Med. Genet. Part A.

[60] Romano A.A., Allanson J.E., Dahlgren J., Gelb B.D., Hall B., Pierpont M.E., Mary E., Roberts A.E., Robinson W., Noonan J.A. (2010). Noonan syndrome: Clinical features, diagnosis, and management guidelines. Pediatrics.

[61] Kratz C.P., Zampino G., Kriek M., Kant S.G., Leoni C., Pantaleoni F., Oudesluys-Murphy A.M., Di Rocco C., Kloska S.P., Tartaglia M. (2009). Craniosynostosis in patients with Noonan syndrome caused by germline KRAS mutations. Am. J. Med. Genet. Part A.

[62] Wessels M.W., Brooks A.S., Hoogeboom J., Niermeijer M.F., Willems P.J. (2002). Kabuki syndrome: A review study of three hundred patients. Clin. Dysmorphol..

[63] McMahon C.J., Reardon W. (2006). The spectrum of congenital cardiac malformations encountered in six children with Kabuki syndrome. Cardiol. Young.

[64] Galvis M.M.O., Bhakta R.T., Tarmahomed A., Mendez M.D. (2023). Cyanotic Heart Disease.

[65] Dundar B., Akcoral A., Saylam G., Unal N., Mese T., Hudaoglu S., Büyükgebiz Β., Böber Ε., Buyukgebiz A. (2000). Chronic hypoxemia leads to reduced serum IGF-I levels in cyanotic congenital heart disease. J. Pediatr. Endocrinol. Metab..

[66] Simmen S., Maane M., Rogler S., Baebler K., Lang S., Cosin-Roger J., Atrott K., Frey-Wagner I., Spielmann P., Wenger R.H. (2021). Hypoxia reduces the transcription of fibrotic markers in the intestinal mucosa. Inflamm. Intest. Dis..

[67] Singhal R., Shah Y.M. (2020). Oxygen battle in the gut: Hypoxia and hypoxia-inducible factors in metabolic and inflammatory responses in the intestine. J. Biol. Chem..

[68] Zeitouni N.E., Chotikatum S., von Köckritz-Blickwede M., Naim H.Y. (2016). The impact of hypoxia on intestinal epithelial cell functions: Consequences for invasion by bacterial pathogens. Mol. Cell. Pediatr..

[69] Obeagu E.I., Mohamod A.H. (2023). An update on Iron deficiency anaemia among children with congenital heart disease. Int. J. Curr. Res. Chem. Pharm. Sci..

[70] Mukherjee S., Sharma M., Devgan A., Jatana S. (2018). Iron deficiency anemia in children with cyanotic congenital heart disease and effect on cyanotic spells. Med. J. Armed Forces India.

[71] Said Y.H., Assenga E., Munubhi E., Kisenge R. (2022). Prevalence of iron deficiency and iron deficiency anaemia among children with congenital heart defects at tertiary hospitals in Dar es Salaam, Tanzania: A cross-sectional study. Pan Afr. Med. J..

[72] Hamano H., Niimura T., Horinouchi Y., Zamami Y., Takechi K., Goda M., Imanishi M., Chuma M., Izawa-Ishizawa Y., Miyamoto L. (2020). Proton pump inhibitors block iron absorption through direct regulation of hepcidin via the aryl hydrocarbon receptor-mediated pathway. Toxicol. Lett..

[73] Shalev H., Quider A.A., Harosh M.B., Kapelushnik J. (2016). Proton pump inhibitors use suppresses iron absorption in congenital dyserythropoietic anemia. Pediatr. Hematol Oncol.

[74] Soliman A.T., Al Dabbagh M.M., Habboub A.H., Adel A., Humaidy N.A., Abushahin A. (2009). Linear Growth in Children with Iron Deficiency Anemia Before and After Treatment. J. Trop. Pediatr..

[75] Okoromah C.A., Ekure E.N., Lesi F.E., Okunowo W.O., Tijani B.O., Okeiyi J.C. (2011). Prevalence, profile and predictors of malnutrition in children with congenital heart defects: A case–control observational study. Arch. Dis. Child..

[76] Rahman M., Dipti D.I., Ahmed M.Y. (2025). Iron Deficiency Anemia among Children with Congenital Heart Disease-A Cross-Sectional Study. Sch. J. Appl. Med. Sci..

[77] Laron Z. (2001). Insulin-like growth factor 1 (IGF-1): A growth hormone. Mol. Pathol..

[78] Fuhrman D.Y., Nguyen L., Joyce E.L., Priyanka P., Kellum J.A. (2021). Outcomes of adults with congenital heart disease that experience acute kidney injury in the intensive care unit. Cardiol Young.

[79] Hamed D.R. (2023). Renal dysfunction in children with congenital cyanotic heart disease. Zagazig Univ. Med. J..

[80] Li S., Krawczeski C.D., Zappitelli M., Devarajan P., Thiessen-Philbrook H., Coca S.G., Kim R.W., Parikh C.R. (2011). Incidence, risk factors, and outcomes of acute kidney injury after pediatric cardiac surgery: A prospective multicenter study. Crit. Care Med..

[81] Morgan C.J., Zappitelli M., Robertson C.M., Alton G.Y., Sauve R.S., Joffe A.R., Ross D.B., Rebeyka I.M. (2013). Risk factors for and outcomes of acute kidney injury in neonates undergoing complex cardiac surgery. J. Pediatr..

[82] Parikh C.R., Devarajan P., Zappitelli M., Sint K., Thiessen-Philbrook H., Li S., Kim R.W., Richard W., Koyner J.L., Coca S.G. (2011). Postoperative biomarkers predict acute kidney injury and poor outcomes after pediatric cardiac surgery. J. Am. Soc. Nephrol..

[83] Lindle K.A., Dinh K., Moffett B.S., Kyle W.B., Montgomery N.M., Denfield S.D., Susan D., Knudson J.D. (2014). Angiotensin-converting enzyme inhibitor nephrotoxicity in neonates with cardiac disease. Pediatr. Cardiol..

[84] Phelps C.M., Eshelman J., Cruz E.D., Pan Z., Kaufman J. (2012). Acute kidney injury after cardiac surgery in infants and children: Evaluation of the role of angiotensin-converting enzyme inhibitors. Pediatr. Cardiol..

[85] Mak R.H., Ikizler A.T., Kovesdy C.P., Raj D.S., Stenvinkel P., Kalantar-Zadeh K. (2011). Wasting in chronic kidney disease. J. Cachexia Sarcopenia Muscle.

[86] Furth S.L. (2005). Growth and nutrition in children with chronic kidney disease. Adv. Chronic Kidney Dis..

[87] Rees L., Mak R.H. (2011). Nutrition and growth in children with chronic kidney disease. Nat. Rev. Nephrol..

[88] Mahesh S., Kaskel F. (2008). Growth hormone axis in chronic kidney disease. Pediatr. Nephrol..

[89] Opotowsky A.R. (2015). Clinical evaluation and management of pulmonary hypertension in the adult with congenital heart disease. Circulation.

[90] Kelleher D.K., Laussen P., Teixeira-Pinto A., Duggan C. (2006). Growth and correlates of nutritional status among infants with hypoplastic left heart syndrome (HLHS) after stage 1 Norwood procedure. Nutrition.

[91] Peterson R.E., Wetzel G.T. (2004). Growth failure in congenital heart disease: Where are we now?. Curr. Opin. Cardiol..

[92] Ross F.J., Radman M., Jacobs M.L., Sassano-Miguel C., Joffe D.C., Hill K.D., Chirswell K., Feng L., Jacobs J.P., Vener D.F. (2020). Associations between anthropometric indices and outcomes of congenital heart operations in infants and young children: An analysis of data from the society of thoracic surgeons database. Am. Heart J..

[93] Hassan B.A., Albanna E.A., Morsy S.M., Siam A.G., Al Shafie M.M., Elsaadany H.F., Sherbiny H.S., Shehab M., Grollmuss O. (2015). Nutritional status in children with un-operated congenital heart disease: An Egyptian center experience. Front. Pediatr..

[94] Woldesenbet R., Murugan R., Mulugeta F., Moges T. (2021). Nutritional status and associated factors among children with congenital heart disease in selected governmental hospitals and cardiac center, Addis Ababa Ethiopia. BMC Pediatr..

[95] Pascall E., Tulloh R.M. (2018). Pulmonary hypertension in congenital heart disease. Future Cardiol..

[96] Diller G.-P., Gatzoulis M.A. (2007). Pulmonary vascular disease in adults with congenital heart disease. Circulation.

[97] Moledina S., Hislop A., Foster H., Schulze-Neick I., Haworth S. (2010). Childhood idiopathic pulmonary arterial hypertension: A national cohort study. Heart.

[98] Alsaied T., Lubert A.M., Goldberg D.J., Schumacher K., Rathod R., Katz D.A., Opotowsky A.R., Jenkins M., Smith C., Rychik J. (2022). Protein losing enteropathy after the Fontan operation. Int. J. Cardiol. Congenit. Heart Dis..

[99] Soliman A., Yassin H., Saeed S., Khellah A., Elalaily R., Elawwa A. (2012). 505 Linear Growth in Children with Congenital Acyanotic Heart Disease (Chd) before Versus after Surgical Intervention. Arch. Dis. Child..

[100] Kubicki R., Grohmann J., Kunz K.-G., Stiller B., Schwab K.O., Van der Werf-Grohmann N. (2020). Frequency of thyroid dysfunction in pediatric patients with congenital heart disease exposed to iodinated contrast media–a long-term observational study. J. Pediatr. Endocrinol. Metab..

[101] Hauck A., Porta N., Lestrud S., Berger S. (2017). The pulmonary circulation in the single ventricle patient. Children.

[102] Coutant R., Bouhours-Nouet N. (2012). Endocrine control and regulation of growth hormone: An overview. Handbook of Growth and Growth Monitoring in Health and Disease.

[103] Soliman A.T., ElAwwa A. (2012). Catch-up growth: Role of GH–IGF-I axis and thyroxine. Handbook of Growth and Growth Monitoring in Health and Disease.

[104] De Staebel O. (2000). Malnutrition in Belgian children with congenital heart disease on admission to hospital. J. Clin. Nurs..

[105] Lewis M.I., Li H., Huang Z.-S., Biring M.S., Cercek B., Fournier M. (2003). Influence of varying degrees of malnutrition on IGF-I expression in the rat diaphragm. J. Appl. Physiol..

[106] Mousikou M., Kyriakou A., Skordis N. (2023). Stress and Growth in Children and Adolescents. Horm. Res. Paediatr..

[107] Kraemer W.J., Ratamess N.A., Hymer W.C., Nindl B.C., Fragala M.S. (2020). Growth hormone (s), testosterone, insulin-like growth factors, and cortisol: Roles and integration for cellular development and growth with exercise. Front. Endocrinol..

[108] Geukers V.G., Li Z., Ackermans M.T., Bos A.P., Jinfeng L., Sauerwein H.P. (2012). High-carbohydrate/low-protein-induced hyperinsulinemia does not improve protein balance in children after cardiac surgery. Nutrition.

[109] Chrousos G. (2000). The role of stress and the hypothalamic–pituitary–adrenal axis in the pathogenesis of the metabolic syndrome: Neuro-endocrine and target tissue-related causes. Int. J. Obes..

[110] Nassef Y.E., Hamed M.A., Aly H.F. (2014). Inflammatory cytokines, apoptotic, tissue injury and remodeling biomarkers in children with congenital heart disease. Indian J. Clin. Biochem..

[111] Zorzanelli L., Maeda N., Clavé M., Thomaz A., Galas F., Rabinovitch M., Lopes A. (2017). Relation of cytokine profile to clinical and hemodynamic features in young patients with congenital heart disease and pulmonary hypertension. Am. J. Cardiol..

[112] Noori N.M., Shahramian I., Teimouri A., Keyvani B., Mahjoubifard M. (2017). Serum levels of tumor necrosis factor-α and interleukins in children with congenital heart disease. J. Tehran Univ. Heart Cent..

[113] Greenhalgh C.J., Alexander W.S. (2004). Suppressors of cytokine signalling and regulation of growth hormone action. Growth Horm. IGF Res..

[114] Cammisa I., Rigante D., Cipolla C. (2025). A Theoretical Link Between the GH/IGF-1 Axis and Cytokine Family in Children: Current Knowledge and Future Perspectives. Children.

[115] Lerner R.K., Gruber N., Pollak U. (2019). Congenital heart disease and thyroid dysfunction: Combination, association, and implication. World J. Pediatr. Congenit. Heart Surg..

[116] Scavone M., Tallarico V., Stefanelli E., Parisi F., De Sarro R., Salpietro C., Ceravolo G., Sestito S., Pensabene L., Chimenz R. (2020). Cardiac malformations in children with congenital hypothyroidism. J. Biol. Regul. Homeost. Agents.

[117] Batra Y.K., Singh B., Chavan S., Chari P., Dhaliwal R.S., Ramprabu K. (2000). Effects of cardiopulmonary bypass on thyroid function. Ann. Card. Anaesth..

[118] Marks S.D., Haines C., Rebeyka I.M., Couch R.M. (2009). Hypothalamic-pituitary-thyroid axis changes in children after cardiac surgery. J. Clin. Endocrinol. Metab..

[119] Saranac L., Zivanovic S., Stamenkovic H., Stankovic T., Djuric Z. (2013). Growth in Children with Thyroid Dysfunction. Current Topics in Hypothyroidism with Focus on Development.

[120] Tarım Ö. (2011). Thyroid hormones and growth in health and disease. J. Clin. Res. Pediatr. Endocrinol..

[121] Fitria L., Caesa P., Joe J., Marwali E.M. (2019). Did malnutrition affect post-operative somatic growth in pediatric patients undergoing surgical procedures for congenital heart disease?. Pediatr. Cardiol..

[122] Weintraub R.G., Menahem S. (1993). Growth and congenital heart disease. J. Pediatr. Child Health.

[123] Barton J., Hindmarsh P., Scrimgeour C., Rennie M., Preece M. (1994). Energy expenditure in congenital heart disease. Arch. Dis. Child..

[124] Toole B.J., Toole L.E., Kyle U.G., Cabrera A.G., Orellana R.A., Coss-Bu J.A. (2014). Perioperative nutritional support and malnutrition in infants and children with congenital heart disease. Congenit. Heart Dis..

[125] Unger R., DeKleermaeker M., Gidding S.S., Christoffel K.K. (1992). Calories count: Improved weight gain with dietary intervention in congenital heart disease. Am. J. Dis. Child..

[126] Menon G., Poskitt E. (1985). Why does congenital heart disease cause failure to thrive?. Arch. Dis. Child..

[127] Nydegger A., Walsh A., Penny D.J., Henning R., Bines J.E. (2009). Changes in resting energy expenditure in children with congenital heart disease. Eur. J. Clin. Nutr..

[128] Lees M.H., Bristow J.D., Griswold H.E., Olmsted R.W. (1965). Relative hypermetabolism in infants with heart disease and undernutrition. Pediatrics.

[129] Trabulsi J.C., Irving S., Papas M., Hollowell C., Ravishankar C., Marino B., Medoff-Cooper B., Schall J.I., Stallings V. (2015). Total energy expenditure of infants with congenital heart disease who have undergone surgical intervention. Pediatr. Cardiol..

[130] Williams R.V., Zak V., Ravishankar C., Altmann K., Anderson J., Atz A.M., Dunbar-Masterson C., Ghanayem N., Lambert L., Lurito K. (2011). Factors affecting growth in infants with single ventricle physiology: A report from the Pediatric Heart Network Infant Single Ventricle Trial. J. Pediatr..

[131] Hansson L., Lind T., Wiklund U., Öhlund I., Rydberg A. (2019). Fluid restriction negatively affects energy intake and growth in very low birthweight infants with haemodynamically significant patent ductus arteriosus. Acta Paediatr..

[132] Herridge J., Tedesco-Bruce A., Gray S., Floh A.A. (2021). Feeding the child with congenital heart disease: A narrative review. Pediatr. Med..

[133] Larson-Nath C., Goday P. (2019). Malnutrition in children with chronic disease. Nutr. Clin. Pract..

[134] Alten J.A., Rhodes L.A., Tabbutt S., Cooper D.S., Graham E.M., Ghanayem N., Marino B.S., Figueroa M.I., Chanani N.K., Jacobs J.P. (2015). Perioperative feeding management of neonates with CHD: Analysis of the Pediatric Cardiac Critical Care Consortium (PC4) registry. Cardiol. Young.

[135] Natarajan G., Anne S.R., Aggarwal S. (2010). Enteral feeding of neonates with congenital heart disease. Neonatology.

[136] Luca A.C., Miron I.C., Mîndru D.E., Curpăn A., Stan R.C., Țarcă E., Luca F.-A., Pădureț A.I. (2022). Optimal Nutrition Parameters for Neonates and Infants with Congenital Heart Disease. Nutrients.

[137] Becker K.C., Hornik C.P., Cotten C.M., Clark R.H., Hill K.D., Smith P.B., Lenfestey R.W. (2014). Necrotizing enterocolitis in infants with ductal-dependent congenital heart disease. Am. J. Perinatol..

[138] Coss-Bu J.A., Hamilton-Reeves J., Patel J.J., Morris C.R., Hurt R.T. (2017). Protein Requirements of the Critically Ill Pediatric Patient. Nutr. Clin. Pract..

[139] Şimşek T., Şimşek H.U., Cantürk N.Z. (2014). Response to trauma and metabolic changes: Posttraumatic metabolism. Turk. J. Surg..

[140] Mehta N.M., Duggan C.P. (2009). Nutritional deficiencies during critical illness. Pediatr. Clin..

[141] Argent A.C., Balachandran R., Vaidyanathan B., Khan A., Kumar R.K. (2017). Management of undernutrition and failure to thrive in children with congenital heart disease in low-and middle-income countries. Cardiol. Young.

[142] Cui Y., Li L., Hu C., Shi H., Li J., Gupta R.K., Liang H., Chen X., Gong S. (2018). Effects and Tolerance of Protein and Energy-Enriched Formula in Infants Following Congenital Heart Surgery: A Randomized Controlled Trial. J. Parenter. Enter. Nutr..

[143] Zhang H., Gu Y., Mi Y., Jin Y., Fu W., Latour J.M. (2019). High-energy nutrition in paediatric cardiac critical care patients: A randomized controlled trial. Nurs. Crit. Care.

[144] Justice L., Buckley J.R., Floh A., Horsley M., Alten J., Anand V., Schwartz S.M. (2018). Nutrition considerations in the pediatric cardiac intensive care unit patient. World J. Pediatr. Congenit. Heart Surg..

[145] Avitzur Y., Singer P., Dagan O., Kozer E., Abramovitch D., Dinari G., Shamir R. (2003). Resting energy expenditure in children with cyanotic and noncyanotic congenital heart disease before and after open heart surgery. J. Parenter. Enter. Nutr..

[146] Davenport M.L., Crowe B.J., Travers S.H., Rubin K., Ross J.L., Fechner P.Y., Gunther D.F., Liu C., Geffner M.E., Thrailkill K. (2007). Growth hormone treatment of early growth failure in toddlers with Turner syndrome: A randomized, controlled, multicenter trial. J. Clin. Endocrinol. Metab..

[147] Mehta N.M., Bechard L.J., Zurakowski D., Duggan C.P., Heyland D.K. (2015). Adequate enteral protein intake is inversely associated with 60-d mortality in critically ill children: A multicenter, prospective, cohort study. Am. J. Clin. Nutr..

[148] Newcombe J., Fry-Bowers E. (2017). A post-operative feeding protocol to improve outcomes for neonates with critical congenital heart disease. J. Pediatr. Nurs..

[149] Panchal A.K., Manzi J., Connolly S., Christensen M., Wakeham M., Goday P.S., Mikhailov T.A. (2016). Safety of enteral feedings in critically ill children receiving vasoactive agents. J. Parenter. Enter. Nutr..

[150] Kim J.-Y., Sarnaik A., Farooqi A., Cashen K. (2022). Contemporary feeding practices in postoperative patients with Congenital Heart Disease. Cardiol. Young.

[151] Reyes A.J., Leary W.P., Lockett C.J., Alcocer L. (1982). Diuretics and zinc. S. Afr. Med. J..

[152] Fritzen R., Davies A., Veenhuizen M., Campbell M., Pitt S.J., Ajjan R.A., Stewart A.J. (2023). Magnesium Deficiency and Cardiometabolic Disease. Nutrients.

[153] Chan B., Woodbury A., Hazelwood L., Singh Y. (2025). Feeding Approach to Optimizing Nutrition in Infants with Congenital Heart Disease. J. Cardiovasc. Dev. Dis..

[154] Jochum F., Moltu S.J., Senterre T., Nomayo A., Goulet O., Iacobelli S., Braegger C., Bronsky J., Cai W., Campoy C. (2018). ESPGHAN/ESPEN/ESPR/CSPEN guidelines on pediatric parenteral nutrition: Fluid and electrolytes. Clin. Nutr..

[155] Anderson B.R., Eckels V.L.B., Crook S., Duchon J.M., Kalfa D., Bacha E.A., Krishnamurthy G. (2020). The risks of being tiny: The added risk of low weight for neonates undergoing congenital heart surgery. Pediatr. Cardiol..

[156] Kopf G.S., Mello D.M. (2003). Surgery for congenital heart disease in low-birth weight neonates: A comprehensive statewide Connecticut program to improve outcomes. Conn. Med..

[157] Knowles R.L., Bull C., Wren C., Wade A., Goldstein H. (2014). Modelling Survival and Mortality Risk to 15 Years of Age for a National Cohort of Children with Serious Congenital Heart Defects Diagnosed in Infancy. PLoS ONE.

[158] Anderson J.B., Kalkwarf H.J., Kehl J.E., Eghtesady P., Marino B.S. (2011). Low weight-for-age z-score and infection risk after the Fontan procedure. Ann. Thorac. Surg..

[159] Hoddinott J., Maluccio J., Behrman J.R., Martorell R., Melgar P., Quisumbing A.R., Ramirez-Zea M., Stein A.D., Yount K.M. (2011). The consequences of early childhood growth failure over the life course. Int. Food Policy Res. Inst. Discuss. Pap..

[160] Medoff-Cooper B., Irving S.Y., Hanlon A.L., Golfenshtein N., Radcliffe J., Stallings V.A., Marino B., Ravishankar C. (2016). The association among feeding mode, growth, and developmental outcomes in infants with complex congenital heart disease at 6 and 12 months of age. J. Pediatr..

[161] Mussatto K.A., Hoffmann R., Hoffman G., Tweddell J.S., Bear L., Cao Y., Tanum J., Brosig C. (2015). Risk factors for abnormal developmental trajectories in young children with congenital heart disease. Circulation.

[162] Stieh J., Kramer H., Harding P., Fischer G. (1999). Gross and fine motor development is impaired in children with cyanotic congenital heart disease. Neuropediatrics.

[163] Roberts J.V., Herman C.P. (2022). The psychology of height: An empirical review. Physical Appearance, Stigma, and Social Behavior.

[164] Young T.J., French L.A. (1996). Height and perceived competence of US presidents. Percept. Mot. Ski..

[165] Kim Y.-J., Kim K.-S. (2004). Body image, self esteem and quality of life in grown-up congenital heart patients. Korean J. Rehabil. Nurs..

[166] Masi G., Brovedani P. (1999). Adolescents with congenital heart disease: Psychopathol. implications. Adolescence.

[167] Csapo M. (1991). Psychosocial adjustment of children with short stature (Achondroplasia): Social competence, behavior problems, self-esteem, family functioning, body image, and reaction to frustrations. Behav. Disord..

[168] Gordon M., Crouthamel C., Post E.M., Richman R.A. (1982). Psychosocial aspects of constitutional short stature: Social competence, behavior problems, self-esteem, and family functioning. J. Pediatr..

[169] Ghaemmaghami Z., Khajali Z., Dalili M., Fotovati Z., Moradian M., Sheikhfathollahi M. (2022). Pubertal status of children with congenital heart disease. Cardiol. Young.

[170] Thomet C., Moons P., Schwerzmann M., Schwitz F. (2023). Development of quality indicators of transfer and transition in adolescents and young adults with congenital heart disease. BMC Health Serv. Res..

